# Ptomaines, Leucomaines and Bacterial Proteids, or the Chemical Factors in the Causation of Disease

**Published:** 1892-12

**Authors:** 


PTOMAINES, LEUCOMAINES AND BACTERIAL PROTEIDS, or the Chemical Factors in the Causation of Disease. By VICTOR C. VAUGHAN, Ph. D., M. D., Professor of Hygiene and Physiological Chemistry in the University of Michigan, and Director of the Hygienic Laboratory; and Frederick G. Novy, Sc., D., M. D., Assistant Professor of Hygiene and Physiological Chemistry in the University Michigan. Second edition, revised and enlarged; 12mo, pp. 391. Philadelphia: Lea Brothers & Co. 1891. Price, cloth, $2.25.

The demand for a second edition of this work, in a short space of time, is a beautiful compliment to the authors that their work has been so thoroughly appreciated, and has supplied the physician and scientist with a much needed volume. Prior to the appearance of the first edition of the book, the worker in the field of ptomaines and leucomaines was obliged to con page after page of journals, in order to secure an idea or a synopsis of the subject. The authors have given to the medical fraternity a volume for which they are truly grateful, and without a study of which they cannot be abreast of the times in the science of medicine.

It is a volume which should be in the library of every physician; a volume which should be carefully read and studied by every medical student and practitioner who desires to be in the vanguard of his profession.

The typography is excellent, and the binding is neat and tasteful. J. A. M.

